# A randomised controlled trial evaluating the effect of a brief motivational intervention to promote breastfeeding in postpartum depression

**DOI:** 10.1038/s41598-021-04338-w

**Published:** 2022-01-10

**Authors:** C. Franco-Antonio, E. Santano-Mogena, S. Chimento-Díaz, P. Sánchez-García, S. Cordovilla-Guardia

**Affiliations:** 1 Nursing Department, Nursing and Occupational Therapy College, Universidad de Extremadura, Cáceres, Spain; 2 Health and Care Research Group (GISyC), Universidad de Extremadura, Cáceres, Spain; 3 Medical and Surgical Therapy Department, Nursing and Occupational Therapy College, Universidad de Extremadura, Cáceres, Spain

**Keywords:** Public health, Health care, Lifestyle modification

## Abstract

Postpartum depression (PPD) is the most frequent psychiatric complication during the postnatal period. According to existing evidence, an association exists between the development of PPD and the maintenance of breastfeeding. A brief motivational intervention (bMI), based on the motivational interview, seems effective in promoting breastfeeding. The objective of this study was to analyse the impact of a bMI aiming to promote breastfeeding on the development of PPD and explore the mediating/moderating roles of breastfeeding and breastfeeding self-efficacy in the effect of the intervention on developing PPD. Eighty-eight women who gave birth by vaginal delivery and started breastfeeding during the immediate postpartum period were randomly assigned to the intervention group (bMI) or control group (breastfeeding education). Randomisation by minimisation was carried out. The breastfeeding duration was longer in the intervention group (11.06 (± 2.94) weeks vs 9.02 (± 4.44), *p* = 0.013). The bMI was associated with a lower score on the Edinburgh Postnatal Depression Scale, with a regression β coefficient of − 2.12 (95% CI − 3.82; − 0.41). A part of this effect was mediated by the effect of the intervention on the duration of breastfeeding (mediation/moderation index β = − 0.57 (95% CI − 1.30; − 0.04)). These findings suggest that a bMI aiming to promote breastfeeding has a positive impact preventing PPD mainly due to its effectiveness in increasing the duration of breastfeeding.

## Introduction

Postpartum depression (PPD) is the most frequent psychiatric complication during the postnatal period^1^, with a reported prevalence ranging between 10 and 15% and even up to 30% depending on the diagnostic criteria used^2–5^. PPD is characterised by a persistent state of mind in mothers accompanied by feelings of sadness, uselessness, or hopelessness and the inability to face and feel joy for the new baby^6,7^. Other symptoms, such as agitation, anxiety, loss of appetite, lack of concentration, fatigue, or suicidal ideation, may appear^6,8^. PPD is associated with alterations in the behaviour of the mother regarding her baby that have a negative impact on the emotional, cognitive, and motor development of the child^4^. In addition, women who suffer from PPD have an increased risk of developing major depression in the future^9^.

The development of this pathology during the postpartum period seems to be related to the hormonal alterations that occur during this period^4^. Other factors that increase the probability of its development have been identified, including (i) a history of psychiatric disorders (history of depression, anxiety or depression in pregnancy or postpartum depression)^7,10^; (ii) stressful life events (including stressors related to childcare), problems in the marital relationship, and lack of support^11,12^; (iii) sociodemographic characteristics (low educational or socioeconomic level, absence of a partner, or unemployment)^7,13^; and (iv) some clinical circumstances, such as caesarean delivery, primiparity, and complications during pregnancy^13,14^.

However, evidence suggests that a relationship exists between breastfeeding (BF) and PPD. Exclusive BF is the ideal food for newborns up to the sixth month of life, and the maintenance of BF along with other foods is recommended for at least 1 or 2 years^15,16^. Optimal BF is associated with benefits to maternal and child health^17–22^, and it has been estimated that the adequate practice of BF is associated with the prevention of 820,000 deaths per year of children aged under 5 years and 19,500 deaths of women related to breast cancer^23^. Therefore, achieving the goal of at least 50% of children aged under 6 months being exclusively breastfed is one of the objectives set by the WHO in its “Global Nutrition Targets 2025”^24^. Regarding the association between BF and PPD, while some authors point out that women with PPD have a shorter duration of BF^25–27^. Other authors note that women who breastfeed are less likely to develop PPD and associate a longer duration of BF with better mental wellbeing among women during the postpartum period and a decrease in the negative feelings that characterise PPD^11,21,28^. Furthermore, several studies have found that mothers with a high level of breastfeeding self-efficacy (BSE) have lower scores on PPD rating scales^29–32^.

### Background

Various approaches to PPD prevention have been implemented as follows: (i) interpersonal counselling interventions ^33,34^, (ii) cognitive-behavioural therapies ^35,36^, (iii) therapies to modify health habits ^37–39^, and (iv) postpartum support interventions ^2,40–44^. A meta-analysis performed for the US Preventive Services Task Force^45^ found that cognitive behavioural therapy and interpersonal therapy are effective interventions for preventing PPD.

Among interpersonal therapies, the brief motivational intervention (bMI) is based on the motivational interview, which could be defined as a collaborative communication style designed to increase personal motivation to achieve one's goals while exploring the individual's ambivalences to change within an atmosphere of acceptance^46^. This type of intervention has already been shown to be effective in increasing the duration of BF and the level of BSE^47–53^. Nevertheless, to the best of our knowledge, only two studies^54,55^ analysed the use of this type of therapy to improve help-seeking when women experience negative feelings associated with PPD.

The evidence suggests that a complex relationship exists among the duration of BF, the presence of PPD^11,21,25,27^, and the level of BSE^29,31^. Therefore, analysing whether an intervention that acts on BF and BSE also has an impact on PPD and determining the causal relationships and the magnitude of the effect among these factors are relevant and pertinent.

## The study

### Aims

This study aimed to analyse the impact of a bMI aiming to promote BF on the development of PPD and explore the mediating/moderating role of BF and BSE in the effect of the intervention on the development of PPD.

For the aims described above, we hypothesise the following:

- Women who receive a bMI would have fewer depressive symptoms in the third month postpartum.
- The effect of the bMI on the BF rates acts as a mediator of the relationship between the bMI and the presence of depressive symptoms postpartum.
- The increase in maternal BSE targeted by the bMI acts as a moderator of the relationship between the bMI and the presence of depressive symptoms postpartum.

### Design

A randomised, controlled, multicentre, parallel-group clinical trial was carried out.

### Participants

The study was carried out in two public hospitals in southwestern Spain between February 2018 and January 2019.

The participants in this study were women who gave birth via vaginal delivery to healthy newborns and who started BF in the hour after delivery regardless of the number of pregnancies or previous deliveries. The exclusion criteria were as follows: (a) mother-newborn separation after delivery due to admission to the neonatal care unit; (b) psychiatric disorder or cognitive damage previously diagnosed in the woman (including the diagnosis of depression before or during pregnancy); (c) impossibility of follow-up with the newborn-mother dyad; (d) women without adequate prenatal care that included prepartum depression screening; and (e) communication barriers.

#### Sample size calculation

The sample size was established based on the effects obtained in similar studies on BF and EBF^48–50^. With a common alpha risk of 0.05, a beta risk of 0.20, and an estimated loss to follow-up of 10%, we calculated that we needed to include 44 participants in each group.

### Procedures

During the immediate postpartum period (first 2 h after delivery), participation in the study was requested from all women who met the inclusion criteria and did not present any of the exclusion criteria based on the clinical history recorded and completed at admission to the hospital until the sample size was reached.

As described in the published study protocol^56^, after recruitment and signing of informed consent, an initial interview was carried out to collect the baseline variables of the participating women. Subsequently, through a randomisation process by minimisation^57^, the women were blindly assigned to one of the two study groups. The midwife responsible for the recruitment and collection of the baseline data was unaware of the randomisation sequence until the investigator in charge of the randomisation reported the result. This researcher obtained the data required for the anonymised randomisation. The collection of the follow‐up data was carried out by another researcher blinded to the intervention/control group status of the participants.

After the randomisation process, the women assigned to the intervention group received a single bMI as follows: a semi-structured interview based on open questions, reflections, and summaries according to the principles of motivational interviewing^46^. The content of the bMI included the exploration of motivations, ambivalences, barriers, and facilitators of the development of BF and the woman's BSE (Table 1).

**Table 1:** Content of the training sessions.

Brief motivational intervention	Breastfeeding educational session
✓ *Introduction:* the objectives of the intervention were explained, and trust was promoted through an empathetic therapeutic approach	✓* A good start to breastfeeding* Skin-to-skin contact and baby-led breastfeeding attachment
✓* Promote self-efficacy:* the woman's confidence in her ability to breastfeed was promoted by identifying the mother's goals, available resources and possible difficulties, promoting learning and reinforcing skills to address BF	✓* Keys to success in breastfeeding in the first days* Keep baby in the room with the mother Responsive feeding Early signs of infant hunger Checking the breastfeeding latch Information regarding stimulating and expressing milk manually or with a breast pump Basic feeding routines and times Avoid teats, dummies and complementary feeds
✓* Motivation:* the woman’s motivation to maintain breastfeeding was explored	✓* Information for the return home* Follow-up of BF by healthcare personnel Useful reference web pages
✓* Exploration of ambivalence:* possible ambivalence was addressed with nonconfrontational responses to the mother's descriptions of any resistance to breastfeeding, her motivation for breastfeeding, and her perceived self‐efficacy	
✓* Final summary:* a verbal review of the most important issues addressed and an opportunity to answer any questions that the mother may have had. Emphasising the advantages of their motivation and ability to find resources and facilitators in their environment to promote their self-efficacy	

The women in the control group received a single educational session that addressed the correct guidelines for achieving successful BF. The session consisted of providing information regarding BF and tips for success (Table 1) using the information leaflet distributed and approved by the Spanish Association of Paediatrics “Congratulations on Your New Motherhood” as a guide^58^.

Both sessions were carried out during the immediate postpartum period in individual sessions by the same midwife with extensive experience in BF who was specifically trained in motivational interviewing by expert psychologists. The duration of the two types of sessions ranged between 20 and 30 min. The women in both groups were given written information regarding BF through the brochure “Congratulations on Your New Motherhood!”^59^ and information regarding BF support groups in their area.

The women in both study groups received booster calls at the 1st postpartum month, lasting no longer than 15 min. The calls in the intervention group followed the principles of the motivational interview, and in the calls to the women in the control group, the women were encouraged to continue BF, and resolutions to possible doubts were offered. At the 3rd month, the women were contacted by phone by another researcher for the collection of the results variables. In both cases, telephone contact was attempted up to 3 times for 1 week before considering the absence of response as a loss of the study.

#### Outcome measures

The variables collected at baseline included the following: (i) sociodemographic variables: age, stable partner (yes/no), educational level (no education, elementary, high school or higher), employment status, and socioeconomic level (< € 13,000/year; € 13,001–21,700/year; and > € 21,700/year); (ii) clinical variables: body mass index, pregnancy weeks, type of delivery, previous children, and newborn weight; and (iii) factors related to BF success: BF experience and BF training. Age, education level, employment situation, and previous experience with BF were the variables used in the randomisation process by minimisation.

As a main variable, the presence of PPD was assessed through the Edinburgh Postnatal Depression Scale (EPDS), a tool that has been translated and validated in Spanish^60^. The EPDS contains questions regarding how the woman has felt during the last 7 days^61^. Women with scores greater than 10 have a high probability of meeting the diagnostic criteria for depression. The EPDS was collected at the third month postpartum. In this study, a score of 11 or higher on the EPDS was considered a cut-off point for the diagnosis of PPD with a sensitivity and specificity of 81 and 88%, respectively^2^.

In this study, information regarding BF was collected at the time of the PPD assessment. Information on the week duration and maintenance of BF (exclusive or not exclusive) was collected in the third month postpartum.

The preintervention and third-month-postpartum BSE levels were also analysed using the Breastfeeding Self-Efficacy Scale Short-Form (BSES-SF)^62^. This 14‐item scale has good internal consistency with a Cronbach's alpha of 0.92. This scale identifies women at the greatest risk of discontinuing BF, assesses BF behaviours and cognitions to individualise confidence‐building strategies, and evaluates the effectiveness of various interventions. This scale has been validated in Spanish.

### Ethical considerations

**Figure 1:**
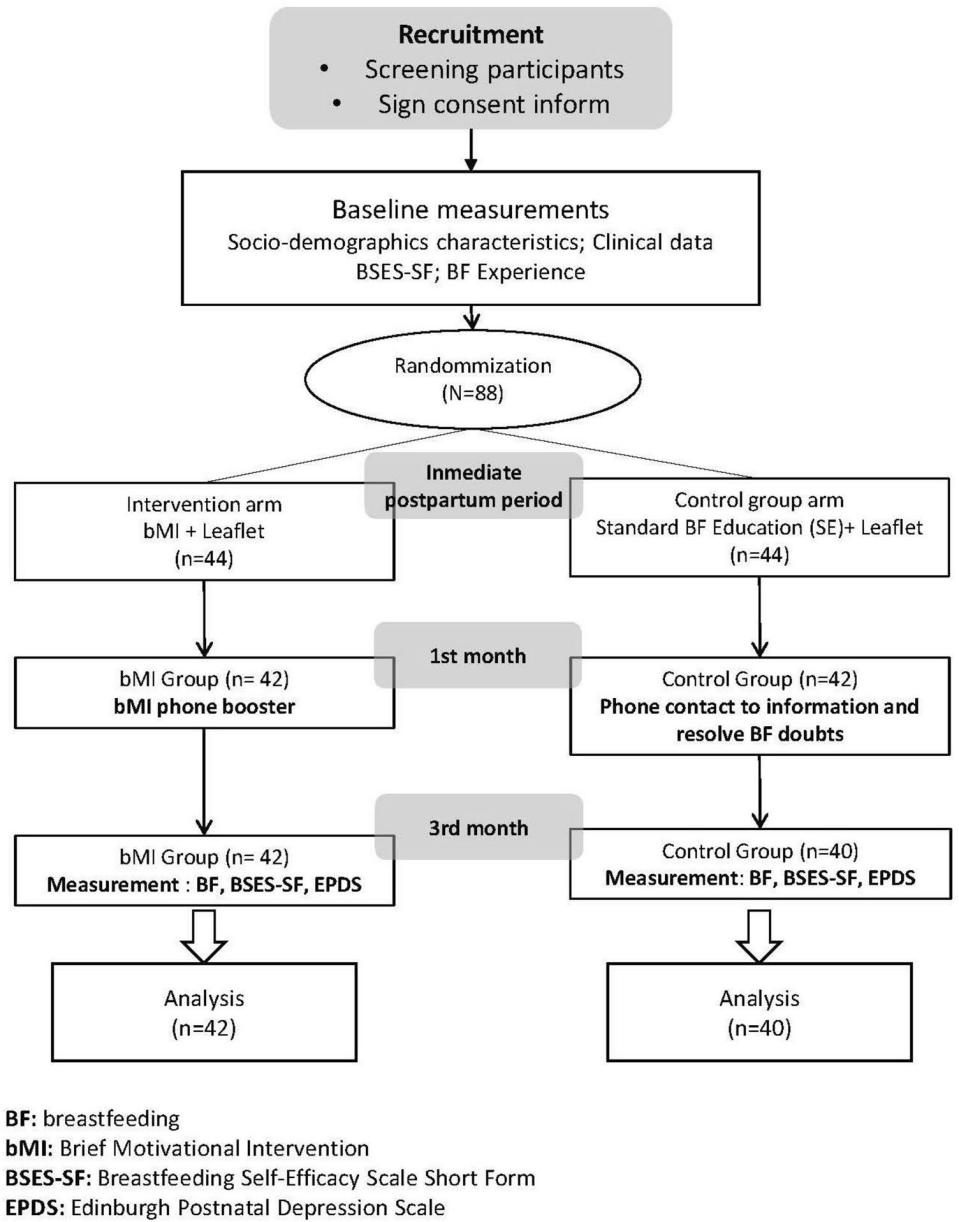
CONSORT diagram of the study.

The Badajoz Clinical Research Ethics Committee of the Extremadura Health Service approved the study in 2017 (180022909). The study protocol was performed in accordance with the relevant guidelines and regulations. The protocol of this study was registered on Clinicaltrials.gov (Identifier: NCT03357549; Date of first registration: 30/11/2017). The participants received written information regarding the aims of the study and were informed that participation was voluntary in accordance with the general recommendations of the Declaration of Helsinki (“World Medical Association Declaration of Helsinki,” 2013). Informed consent was obtained from all participants. The confidentiality of the participants was maintained at all times. Figure 1 shows the CONSORT diagram of the study.

The women participating in this study who were diagnosed with PPD by the EPDS were referred to psychiatric services for proper care.

### Data collection

The baseline variables were collected from the clinical history and initial interview before the randomisation of the participants to each study group. The data from the third month were collected by telephone calls between May 2018 and January 2019. Figure 1 shows the CONSORT diagram of the study.

### Validity and reliability

The EPDS validated in Spanish women has a positive predictive value of 63.2% and a negative predictive value of 97.7%. The Spanish version of the BSES-SF has good internal consistency with a Cronbach’s alpha coefficient of 0.91. To minimise the possibility of bias due to unmeasured characteristics related to the use of different interviewers, the same midwife conducted the bMI and the BF education sessions. Due to randomisation, all participants had an equal chance of receiving the intervention.

### Data analysis

A similar distribution of the baseline characteristics was verified. The quantitative variables were compared between the groups with a Student’s t-test or Mann–Whitney U test according to the normality of their distribution. A Pearson chi-squared test and Fisher's exact test were used for the comparisons of the categorical variables.

To analyse the effect of the intervention on the development of PPD, the scores on the EPDS scale were compared between both study arms. To quantify the strength of the association between the bMI and the development of PPD, a multinomial logistic regression was performed, which allowed us to obtain estimates of the odds ratio (OR) and adjusted OR (aOR) when introducing the maintenance of BF at the time of PPD assessment as an independent variable in the model. All estimates were conducted with their corresponding 95% confidence intervals (95% CIs). All statistical procedures were performed using IBM SPSS Statistics for Windows version 24.0 (IBM, Armonk, New York).

Finally, to determine the role of the increase in BSE and duration of BF in the association between bMI and PPD, mediation and moderation analyses were performed according to the Hayes method^63,64^ through the PROCESS macro^64^ 2016 version in IBM SPSS Statistics for Windows version 24.0 (IBM, Armonk, New York).

## Results

Participation was requested from 88 women who met the inclusion criteria, and none of them declined to participate. During the follow-up period, in total, six losses occurred, all of which were due to the impossibility of phone contact (Fig. 1). These women were somewhat younger, with a mean age (± standard deviation) of 27.83 (± 8.04) years vs. 33.18 (± 4.76) years among those who completed the study (*p* = 0.013); had a lower level of income (income < € 13,001/year: 66.7% vs. 9.2%, *p* = 0.002); and were mostly first-time mothers (83.3% vs. 37.8%, *p* = 0.016).

The mean age of the women was 32.82 years (± 5.17). More women in the intervention group had higher education (34.1% vs 18.2%) and previous experience with BF (63.6% vs 56.8%). No differences were found in the other baseline variables. There were no differences in the baseline score on the BSES-SF as follows: 59.14 (± 9.35) in the bMI group and 59.41 (± 8.45) in the control group (*p* = 0.886) (Table 2).

**Table 2:** Sociodemographic and clinical characteristics of the sample.

	Total sample N = 88	bMI group n = 44	Control group n = 44
Age (years) *M (SD)*	32.82 (5.17)	32.4 (5.99)	33.25 (4.21)
BMI median [IQR]	22.32 [20.25–26]	22.23 [20.66–24.86]	22.67 [19.89–27.08]
**Education *n* (%)**			
No studies/primary	29 (33)	11 (25)	18 (40.9)
Secondary	36 (40.9)	18 (40.9)	18 (40.9)
University/postgraduate	23 (26.1)	15 (34.1)	8 (18.2)
**Employment *n* (%)**			
Unemployed	37 (42)	18 (40.9)	19 (43.2)
Work for others	46 (52.3)	25 (56.8)	21 (47.7)
Own-account work	5 (5.7)	1 (2.3)	4 (9.1)
**Annual family income n (%)**			
< 13,000 €	12 (13.6)	7 (15.9)	5 (11.4)
13,000–21,700 €	61 (69.3)	29 (65.9)	32 (72.7)
> 21,700 €	15 (17)	8 (18.2)	7 (15.9)
Has more children *n *(%)	58 (65.9)	29 (65.9)	29 (65.9)
Previous experience BF *n *(%)	53 (60.2)	28 (63.6)	25 (56.8)
Received specific training in BF *n* (%)	38 (43.2)	19 (43.2)	19 (43.2)
Pregnancy weeks M (SD)	39.14 (1.24)	39.16 (1.18)	39.11 (1.32)
Newborn weight (grams) M (SD)	3270.6 (451.82)	3241.14 (410.58)	3300 (492.63)
**Type of delivery *n* (%)**			
Spontaneous	81 (92)	40 (90.9)	41 (93.2)
Forceps	5 (5.7)	2 (4.5)	3 (6.8)
Vacuum	2 (2.3)	2 (4.5)	0 (0)
BSES-SF Score M (SD)	59.27 (8.86)	59.14 (9.35)	59.41 (8.45)

*M* mean, *SD* standard deviation, *IQR* interquartile range, *bMI* brief motivational intervention, *BF* breastfeeding, *BSES-SF* Breastfeeding Self-Efficacy Scale Short Form, *BMI* body mass index.

The duration of BF (including both exclusive and nonexclusive) was 11.06 (± 2.94) weeks in the bMI group compared to 9.02 (± 4.44) weeks in the control group (*p* = 0.013). In the mothers who completed the follow-up, the baseline BSES-SF scores were practically the same in both groups. However, the intervention group had 4.13 points more than the intervention group at the third month postpartum (Table 3). When comparing the intra-group scores, we found that in the intervention group, the self-efficacy score was increased by 6.24 points, while in the control group, there were no significant differences.

**Table 3:** Intra-group and inter-group comparison of breastfeeding self-efficacy.

	bMI group n = 42	Control group n = 40	*p* value (inter-group)
BSES-SF Basal Score Mean (SD)	59.14 (9.31)	59.68 (8.66)	0.790^1^
BSES-SF 3rd month Score Mean (SD)	65.38 (6.23)	61.25 (8.74)	0.015^1^
*p* value (intra-group)	< 0.001^1^	0.304^1^	

*bMI* brief motivational intervention, *BSES-SF* Breastfeeding Self-Efficacy Scale Short Form.

^1^Student’s t-test.

**Figure 2:**
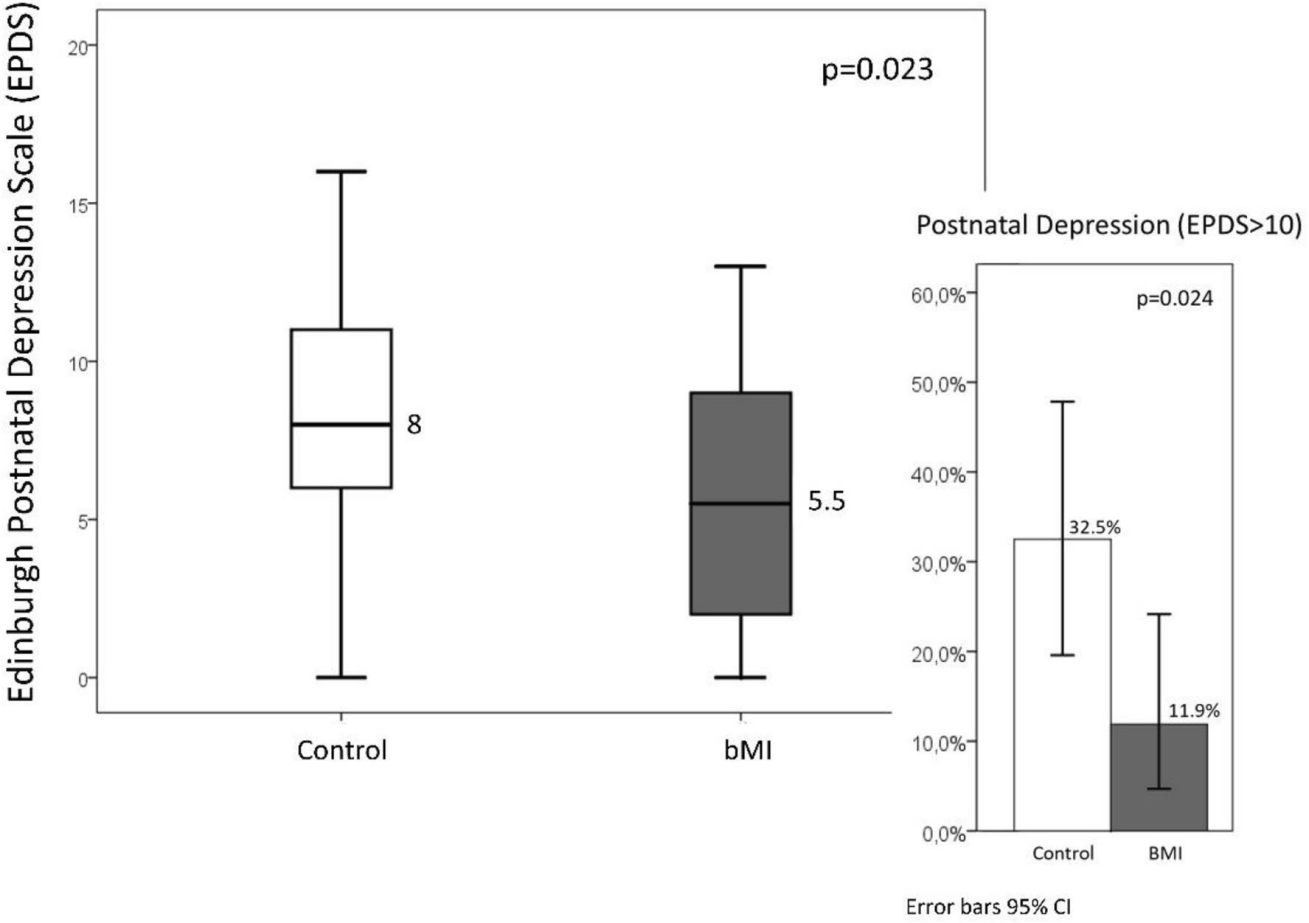
Analysis of postpartum depression in the study groups.

The EPDS score in the control group, with a median [interquartile range] of 8 [6–11], was higher than that in the group that received the bMI, with a median of 5.5 [1.75–9] (Fig. 2). When considering scores above 10, which indicate a high probability of a PPD diagnosis, the percentage of women with a probable diagnosis of PPD was higher in the control group (30% vs. 11.9%) (Fig. 2). When the magnitude of the association was measured, we found that the women who received the intervention were less likely to score above 10 on the EPDS, with an OR of 0.32 (95% CI 0.1; 0.99) (*p* = 0.050). This association magnitude hardly varied when adjusting this effect for BF at the time of the evaluation (aOR of 0.33 [95% CI 0.10; 1.08] *p* = 0.068).

**Figure 3:**
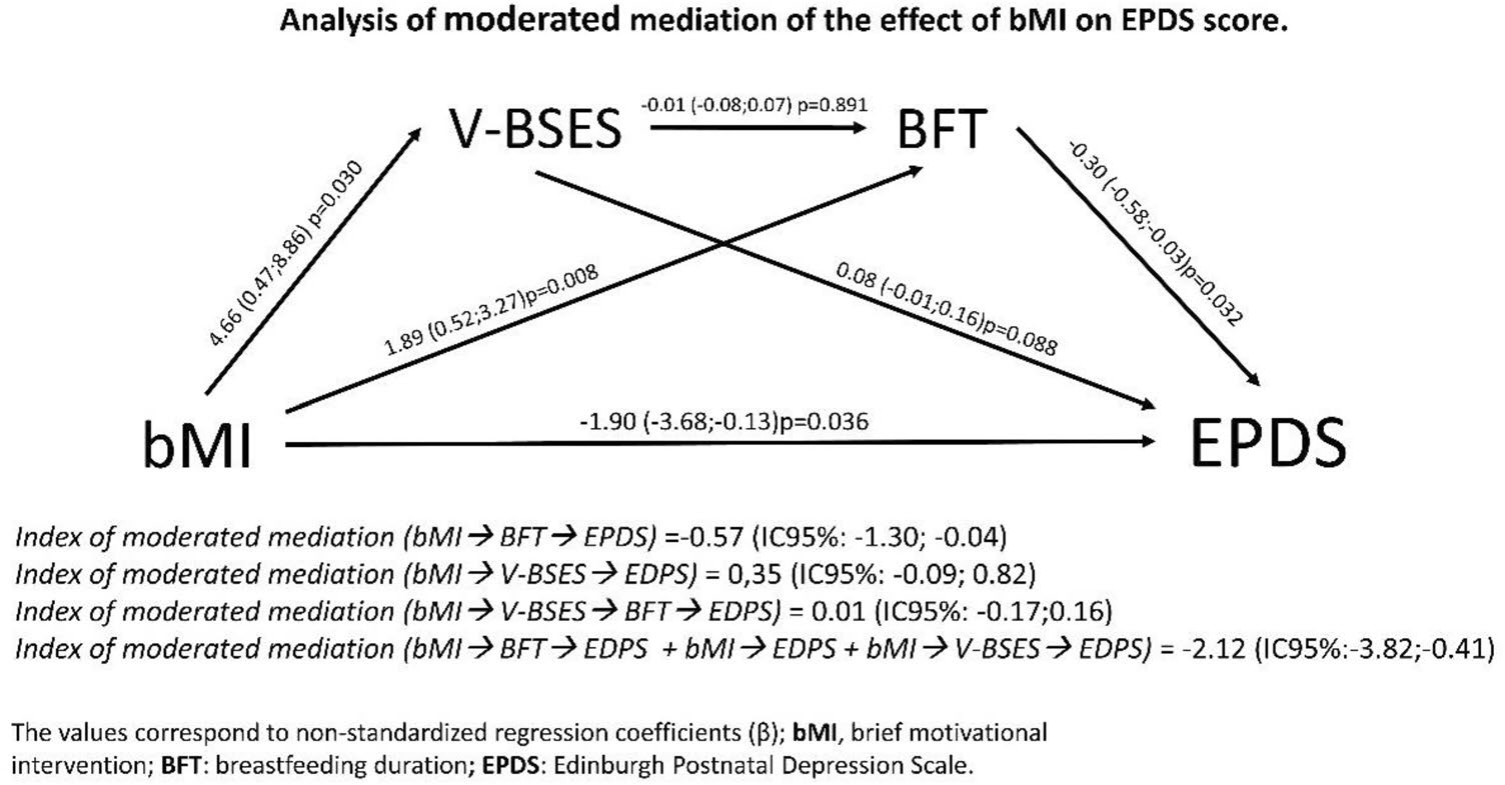
Analysis of the moderated mediation of the effect of the brief motivational intervention on the Edinburgh Postnatal Depression Scale.

The mediation/moderation analyses showed that the effect of the bMI on the variation in BSE did not mediate the effect of the intervention on the score on the EDPS scale, with a β mediation/moderation index of 0.35 (95% CI − 0.09; 0.82) (Fig. 3). However, this mediation/moderation analysis showed that the effect of the intervention on the BF time acted as a mediator of the effect of bMI on the EPDS score, with a β moderation/mediation index of − 0.57 (95% CI − 1.30; − 0.04) (Fig. 3).

The interaction analyses showed that educational level and previous experience with BF did not interfere with the effect of the bMI on the EPDS score (F = 0.672, *p* = 0.514; and F = 1.541, *p* = 0.218, respectively). In addition, analysis of the possible moderating effect of the increase in BSE on the effect of the bMI on PPD showed that it did not act as a moderator as follows: it had a β mediation/moderation index of − 0.25 (95% CI − 0.20; 0.16) (*p* = 0.786).

## Discussion

Our results show that women who received a bMI to promote BF during the immediate postpartum period with telephone reinforcement at 1 month postpartum scored lower on the EPDS at the third postpartum month than those who received an educational session concerning BF at the same time. Our analyses show that the women who received bMI had a lower probability of scoring above 10, which is a score associated with a high probability of the existence of PPD. This probability is slightly modified when considering the maintenance of BF at the time of the PPD assessment.

The existing evidence concerning effective interventions for the prevention of PPD has been analysed in several systematic reviews^65–67^. In a 2013 systematic review^65^, it was found that postpartum visits by nurses or midwives, telephone peer support, and interpersonal psychotherapy were effective in reducing symptoms associated with depression. Furthermore, a recent meta-analysis^66^ found that cognitive behavioural therapy and interpersonal therapy were effective interventions to prevent PPD. The bMI can be classified as an interpersonal therapy^65^, which could explain the effect of the intervention on the EPDS score.

To better understand the results obtained by the bMI on the development of feelings associated with PPD, mediation/moderation analyses were carried out, which showed that a part of the effect of the bMI on the EPDS score was mediated by the effect of the bMI on the duration of BF. The women in the intervention group had a longer duration of BF, which has been associated with a lower probability of developing PPD in some studies^11,21,28^. Although the evidence concerning the association between BF and PPD is unclear, some authors note that women with PPD have a shorter duration of BF^25–27^, and other authors note that women who breastfeed are less likely to develop PPD^11,21,28^. The mediating effect of BF duration on the relationship between bMI and EPDS scores found in our study reinforces the hypothesis of these authors, who found a protective effect of BF on PPD^23,68^. Moreover, in our study, the previous presence of depressive symptoms was ruled out. However, a large part of the effect of bMI on PPD is due exclusively to the intervention. A bMI aiming to promote breastfeeding could indirectly act in the development of negative feelings associated with depression, producing a protective effect^34^. Similar interventions based on motivational interviewing and Bandura's theory of self-efficacy have found that there is an increase in the ability to seek help by women and a decrease in the risk of developing PPD during the postpartum period^34,54^.

Additionally, the applied bMI achieved a greater increase in BSE than the control intervention, and a higher level of BSE has been associated with a lower EPDS score^29–32^. However, the mediation/moderation analyses showed that although the bMI produced a greater increase in BSE than the control intervention, this increase did not mediate the effect that bMI had on developing PPD. These analyses indicate that the bMI also does not moderate the effect of developing PPD. The reason for this discrepancy with previous evidence could be that a higher BSE is also associated with a longer duration of BF^69–72^, and the latter is truly related to the development of PPD.

Therefore, the bMI applied in this study is effective in preventing the development of PPD partially due to the intervention and partially due to its effect on the duration of BF. To our knowledge, this is the first to analyse the impact of an intervention directly aiming to provide BF support on the development of PPD using mediation/moderation analyses. This approach allowed us to estimate the direct and indirect causal effect paths of the intervention on the EPDS score. The role as mediator of BF that we observed in our results allows us to suppose that other interventions aiming to increase the duration of BF could have the same collateral effect on the psychological well-being of women. However, more research is needed to clarify whether other interventions aiming to promote BF could have the same effect on PPD. Furthermore, although the bMI appears to be effective in promoting BF^47–50^, additional studies are still needed to analyse this efficacy in different situations. If these results are confirmed, the convenience of including this type of intervention in routine healthcare practice could be reinforced. The bMI applied in this study is easily integrated into clinical practice. The application of this intervention by nursing and midwifery through its integration into the usual care of mothers and babies could be feasible and easily achievable since it only requires an investment in the specific training of professionals^73^. Increasing the duration of BF could have an important impact on public health due to the associated benefits, such as the reduction in infant and female morbidity and mortality^22,23^, to which could be added a reduction in the burden of disease associated with PPD. This approach could make the widespread implementation of programs to promote BF through bMI a cost–benefit practice, as has occurred in other clinical settings^73^.

### Limitations

The findings of this study are limited by its design since the intervention was applied in women without a history of certain psychiatric disorders (depression, depression and anxiety during pregnancy, or postpartum depression)^7,10^. Such history is one of the most relevant risk factors for the development of PPD; thus, the effectiveness of this intervention could be diminished in women with a psychiatric history. Furthermore, an assessment of baseline EPDS score was not carried out in this study. Although the program of medical check-ups to follow up the pregnancy of the national health system contemplates depression symptoms evaluation, the determination of depression through the clinical history may not have been effective in all cases. Additionally, it was not applied in women who gave birth by caesarean section or had complications in pregnancy, which are two other factors associated with PPD^13,14^. Therefore, it could be necessary to investigate whether this bMI aiming to promote BF has an impact on the development of PPD in women with these characteristics. Finally, this intervention aimed to promote BF; thus, given its design, it would not be feasible to use this bMI to help prevent PPD in women who are unwilling or unable to engage in BF. A modification in the design of the bMI would be required to adapt it to preventing the development of PPD specifically.

Despite our efforts to achieve a balanced distribution of the variables strongly associated with the outcome using randomisation by minimisation, between-group baseline differences in education level and previous experience with BF were found. Although these differences were not very important, these factors were introduced into the adjusted models to prevent their effect on the results. Furthermore, some characteristics of the women classified as lost to follow-up, such as primiparity or low socioeconomic status, are associated with a greater probability of developing PPD. More studies are needed to evaluate how these factors could affect the possible implementation of intervention programmes.

## Conclusions

A bMI for the promotion of BF applied during the immediate postpartum period in women who initiate BF in the first hour of life of the newborn and reinforced by one telephone call per month is associated with a lower score on the EPDS at the third postpartum month.

Part of the effect of this bMI on the EPDS score is due to the intervention itself. However, a significant part of the effect is mediated by the effect of the bMI on the duration of BF, which could support evidence associating a longer duration of BF with a lower probability of PPD development^11,21,28^.

Given the importance of both improving the duration of BF and preventing PPD, more research is needed to support the findings found in this study. In addition, future studies should analyse the efficacy of the bMI in women with greater susceptibility to the development of PPD, such as women with a history of psychiatric disorders, those who deliver by caesarean section, and those with complications during pregnancy.
